# Salvinorin A, a kappa-opioid receptor agonist hallucinogen: pharmacology and potential template for novel pharmacotherapeutic agents in neuropsychiatric disorders

**DOI:** 10.3389/fphar.2015.00190

**Published:** 2015-09-08

**Authors:** Eduardo R. Butelman, Mary Jeanne Kreek

**Affiliations:** Laboratory on the Biology of Addictive Diseases, The Rockefeller University, New York, NY, USA

**Keywords:** kappa-opioid receptor, dynorphins, salvinorin A, *Salvia divinorum*, depression, addiction

## Abstract

Salvinorin A is a potent hallucinogen, isolated from the ethnomedical plant *Salvia divinorum.* Salvinorin A is a selective high efficacy kappa-opioid receptor (KOPr) agonist, and thus implicates the KOPr system and its endogenous agonist ligands (the dynorphins) in higher functions, including cognition and perceptual effects. Salvinorin A is the only selective KOPr ligand to be widely available outside research or medical settings, and salvinorin A-containing products have undergone frequent non-medical use. KOPr/dynorphin systems in the brain are known to be powerful counter-modulatory mechanisms to dopaminergic function, which is important in mood and reward engendered by natural and chemical reinforcers (including drugs of abuse). KOPr activation (including by salvinorin A) can thus cause aversion and anhedonia in preclinical models. Salvinorin A is also a completely new scaffold for medicinal chemistry approaches, since it is a non-nitrogenous neoclerodane, unlike other known opioid ligands. Ongoing efforts have the goal of discovering novel semi-synthetic salvinorin analogs with potential KOPr-mediated pharmacotherapeutic effects (including partial agonist or biased agonist effects), with a reduced burden of undesirable effects associated with salvinorin A.

## Salvinorin A

Salvinorin A (derived from the ethnomedical plant *Salvia divinorum*) is a powerful hallucinogen in humans, and is a selective, high efficacy agonist at kappa-opioid receptors (KOPr; Roth et al., 2002; Chavkin et al., 2004). Salvinorin A is unique for several reasons, which include: (a) it is the first plant-derived ligand with high selectivity for KOPr over other receptors, including mu-opioid receptor (MOPr) (the target of opioid alkaloids, such as morphine), (b) it is structurally unrelated to any known opioid receptor ligand, and is a non-nitrogenous diterpene, (c) it is pharmacologically and mechanistically distinct from other known hallucinogens in humans (e.g., classic serotonergic hallucinogens, which are primarily 5HT2A agonists), (d) it is the only selective KOPr ligand to become relatively widely available outside research or medical settings. This is also related to its diffusion through the internet and other commercial outlets, due to its complex legal status across jurisdictions.

## The KOPr System and its Endogenous Agonist Neuropeptides, the Dynorphins

KOPr receptors are members of the opioid receptor 7-transmembrane domain (7TM) G-protein coupled receptor (GPCR) superfamily, coupled primarily to G_*i/o*_ proteins that inhibit actions of adenylyl cyclase, and also activate alternate downstream pathways, including β-arrestin-mediated pathways and ERK/MAP kinase pathways (Dhawan et al., 1996; Bruchas and Chavkin, 2010; Zhou et al., 2013a). The endogenous KOPr agonists are the dynorphin neuropeptides, encoded by gene *PDYN* in humans (Chavkin and Goldstein, 1981; Chavkin, 2012). KOPr and the dynorphins are located in many parts of the central nervous system (CNS; and also in the peripheral nervous system). In the CNS, KOPr/dynorphin systems are prominent in the dorsal and ventral striatum (e.g., caudate-putamen and Nucleus accumbens, respectively), several cortical areas, and limbic areas including hippocampus and amygdala, as well as hypothalamus and spinal cord (Mansour et al., 1988; Spangler et al., 1993; Unterwald et al., 1994; Simonin et al., 1995; Mathieu-Kia et al., 2001). Thus, this receptor/ligand system mediates diverse behavioral functions, including mediation of reward and aversion, mood, anxiety, stress-responsivity, memory and higher cognitive functions, as well as neuroendocrine effects and pain/analgesia. KOPr/dynorphin systems are co-localized in most major dopaminergic areas in the CNS. KOPr activation, by exogenous ligands including salvinorin A, or by dynorphins, tends to result in a decrease in dopaminergic activation in dorsal and ventral striatum (Di Chiara and Imperato, 1988; Spanagel et al., 1990; Zhang et al., 2004, 2005; Carlezon et al., 2006; i.e., an effect opposite to that of diverse drugs of abuse, including cocaine, other psychostimulants and MOPr agonists).

## Effects of KOPr Activation on Behavioral and Neurobiological Endpoints

Based on preclinical studies in rodents and non-human primates, and in human Laboratory studies, activation of KOPr is thought to result in a variety of robust effects summarized below: (a) decreases in locomotor activity and arousal, also manifested as sedative-like behaviors, (b) dysphoria and aversion, (c) depressant-like behaviors and (d) modulation in the effects of drugs of abuse (e.g., in conditioned place preference or self-administration studies). Of interest, in the Laboratory—based human research studies to date (carried out in experienced hallucinogen users), salvinorin A did not result in robust dysphoric or aversive subjective effects (in the presence of other robust subjective effects, including hallucinations; Johnson et al., 2011; Addy, 2012; Ranganathan et al., 2012; MacLean et al., 2013). It is unknown if this is due to experimental context, subject population characteristics, or to a differential pharmacodynamic profile of salvinorin A, compared to synthetic KOPr agonists used in other studies in humans (Pfeiffer et al., 1986; Pande et al., 1996). For example, it is unknown if repeated prior exposure to salvinorin A in the recruited subjects resulted in relative development of neuroadaptations, including tolerance, in KOPr populations.

## Status of the KOPr/dynorphin System During Stress, or in Addictive States

Notably, endogenous activation of KOPr caused by stress can result in several of the aforementioned types of behaviors (e.g., depressant-like behaviors), primarily by increases in dynorphin actions, in specific neuroanatomical areas (Zhang et al., 2007; Reed et al., 2012; Cohen et al., 2014). Stress exposure can also result in relapse-like behaviors in preclinical models, in part mediated by endogenous KOPr/dynorphin activation (Beardsley et al., 2005; Aldrich et al., 2013; Zhou et al., 2013b). Also, exposure to drugs of abuse (e.g., cocaine, other psychostimulants or short-acting MOPr agonists, such as morphine) results in an upregulation in the KOPr/dynorphin system, as measured either by KOPr autoradiography, by signaling assays, or by PDYN mRNA (Daunais et al., 1993; Spangler et al., 1993; Unterwald et al., 1994; Wang et al., 1999; Adams et al., 2003; Whitfield et al., 2015). It has been thus hypothesized that several neuropsychiatric conditions including stress-induced depressant or anxiety-like effects, as well as escalation of drug intake, and relapse-like behaviors are mediated in part by KOPr/dynorphin upregulation (Spangler et al., 1993; Nestler and Carlezon, 2006; Bruchas et al., 2010; Chavkin, 2011; Butelman et al., 2012b).

Given the diverse and robust effects of KOPr activation in these behavioral and neurobiological endpoints, a considerable amount of recent work (mostly preclinical), has focused on acute and chronic effects of salvinorin A exposure.

## *In vivo* Effects of Salvinorin A

Salvinorin A causes overt sedative-like and locomotor-decreasing effects in rodent and non-human primate models (including unresponsiveness to environmental stimuli; Fantegrossi et al., 2005; Zhang et al., 2005; Butelman et al., 2009). These effects are qualitatively similar to those of synthetic KOPr agonists, and are sensitive to KOPr antagonism (Fantegrossi et al., 2005; Zhang et al., 2005; Butelman et al., 2009). Hallucinogenic effects *per se* are difficult to model in a valid manner in non-human models, since they depend on interoceptive and subjective states that are not easily discernible by behavioral observation in non-verbal species.

## Drug Discrimination

Data from non-human primate and rodent models show that discriminative effects salvinorin A (potentially modeling interoceptive effects) are mediated by agonist effects at KOPr, and not by 5HT2A receptors, site of action of most classic hallucinogens (such as LSD and psilocybin; Roth et al., 2002; Butelman et al., 2004b, 2010; Li et al., 2008; Baker et al., 2009; Killinger et al., 2010). Recent studies in humans also show that naltrexone (50 mg, p.o.) blocked the effects of salvinorin A in humans (Valle et al., 2014). This is consistent with blockade of KOPr agonist effects of salvinorin A, since sufficient doses of naltrexone can robustly block the effects of diverse KOPr ligands in human and non-human primates (these naltrexone doses are usually larger than doses minimally required to block MOPr mediated effects; Ko et al., 1998; Butelman et al., 2004a; Walsh et al., 2008). Overall these data implicate the KOPr/dynorphin system as a mechanism mediating several higher functions, including mood, sedation and arousal, as well as depressant-like effects, perception and higher cognitive functions.

## Neuroendocrine Effects

KOPr agonists have specific neuroendocrine effects, including prolactin release and also stimulation of HPA axis hormones, ACTH and cortisol (or corticosterone in rodents; Adamson et al., 1991; Ur et al., 1997; Kreek et al., 1999; Pascoe et al., 2008). These effects are thought to be primarily mediated by KOPr at different hypothalamic sites, including arcuate and paraventricular nuclei, although this has not been unequivocally confirmed.

Prolactin has been used as a simple quantitative biomarker of KOPr agonist effects, and it can be used to discern onset, potency (e.g., ED_50_) and apparent efficacy of KOPr ligands (Butelman et al., 1999; Kreek et al., 1999; Bart et al., 2005; Chang et al., 2011; other compounds, including MOPr agonists, also cause prolactin release). Salvinorin A was found to be a potent efficacious, and rapid-onset KOPr agonist in this biomarker in non-human primates with high KOPr homology to humans (Butelman et al., 2007). Recent work in humans also reported similar effects (see below; Ranganathan et al., 2012).

## Salvinorin A Effects in Preclinical Assays for Neuropsychiatric Diseases (e.g., Mood, Anxiety, and Addictions)

Similarly to synthetic KOPr agonists and to endogenous KOPr ligands (dynorphins), salvinorin A causes a dose-dependent decrease in dopamine dialysates in dorsal and ventral striatum of rodents (Zhang et al., 2005; Carlezon et al., 2006); these effects occur at doses similar to those that cause behavioral effects such as place aversion, depressant-like effects, and locomotor decreases. Neurobiological studies suggest that one possible mechanism of the decreases in dopamine levels may be a salvinorin A-induced potentiation of dopamine re-uptake transporter (DAT) function, and this may be secondary to the formation of KOPr/DAT heterodimers (Kivell et al., 2014). Acute and chronic effects of salvinorin A on dopaminergic systems may also differ (Gehrke et al., 2008), of possible relevance to repeated users of salvinorin A-containing products. Paradoxically, some studies have reported that relatively smaller salvinorin A doses cause a detectable *enhancement* of dopamine dialysate levels (Braida et al., 2008; Serra et al., 2015). The differential mechanisms involved in this latter effect have not been fully elucidated.

Salvinorin A, similarly to synthetic KOPr agonists, results in dose-dependent conditioned aversion (e.g., in contextual conditioning assays; Zhang et al., 2005). Also, salvinorin A can act as a “punisher” to self-administration of cocaine or a short-acting MOPr agonist in primates (when given contingently; Whitfield et al., 2015). These studies would appear to be discrepant with the relatively wide experimentation observed with salvinorin A-containing products in humans. However, it should be noted that classic serotonergic hallucinogens self-administered by humans do not produce stable or robust reinforcing effects in preclinical studies (Fantegrossi et al., 2004). Furthermore, self-administration of hallucinogens in humans tends to follow a more episodic nature than that of other drugs of abuse, including cocaine or MOP-r agonists, and may occur in specific subsets of the human population that find hallucinogens rewarding or attractive.

Salvinorin A also results in anhedonia in ICSS assays and depressant-like effects in the forced swim test, similarly to synthetic KOPr agonists (Carlezon et al., 2006; Negus et al., 2012). KOPr antagonists, by contrast, can block these salvinorin A-induced effects, and alone also produce anti-depressant like-effects, and anxiolytic-like effects (Carlezon et al., 2006; Negus et al., 2012; Reed et al., 2012; Rorick-Kehn et al., 2014). These findings lead to the postulation that prolonged high efficacy signaling through KOPr results in behavioral and neurobiological effects associated with human neuropsychiatric conditions, especially depression-like and anxiety-like states, and specific addictions. Conversely, novel compounds with limited efficacy at KOPr (such as antagonists or appropriate partial agonists) or compounds with “biased” KOPr signaling, could potentially have pharmacotherapeutic effects in mood, anxiety or addictive states (see below; Neumeyer et al., 2001; Butelman et al., 2012b; Tejeda et al., 2012; Bidlack, 2014; Simonson et al., 2014; Lovell et al., 2015; Whitfield et al., 2015).

## Studies of Salvinorin A and *Salvia divinorum* in Humans

There have been initial descriptive and ethnographic reports of *Salvia divinorum* or salvinorin A exposure (Valdes et al., 1983; Siebert, 1994). More recently several controlled laboratory-based studies have characterized the *in vivo* effects of salvinorin A or *Salvia divinorum* in humans (Johnson et al., 2011; Mendelson et al., 2011; Addy, 2012; Ranganathan et al., 2012; MacLean et al., 2013). There have also been several questionnaire-based studies and clinical case studies of the effects of salvinorin A-containing preparations in humans (Singh, 2007; Lange et al., 2008; Przekop and Lee, 2009; Baggott et al., 2010; Wu et al., 2011; Perron et al., 2012).

Carefully controlled studies in humans, initially experienced hallucinogen or *Salvia divinorum* users, characterized the effects of salvinorin A smoking (0.75–21 μg/kg). Salvinorin A’s effects were of extremely rapid onset, peaked by 2 min after inhalation, and declined by 30 min (Johnson et al., 2011; MacLean et al., 2013). Under these carefully monitored conditions, volunteers reported robust hallucinogenic-like effects, depersonalization and derealization, but no robust dysphoria or aversion (Johnson et al., 2011). It is unknown if effects in a different population (i.e., non-hallucinogen users) would show a more robust dysphoria/aversion signal, consistent with effects observed in preclinical rodent models (Zhang et al., 2005; Carlezon et al., 2006), or dysphoric effects reported with synthetic KOPr agonists in humans (Kumor et al., 1986; Pfeiffer et al., 1986; Pande et al., 1996; Walsh et al., 2001). A separate study with smoked salvinorin A (in volunteers with previous self-exposure to *Salvia divinorum*) reported dose-dependent and reversible psychotomimetic effects, dissociation, and neuroendocrine effects (increases in serum cortisol and prolactin; Ranganathan et al., 2012). A further study examined the effects of *Salvia divinorum* smoking, and characterized subjective experiences including cognitive alterations, which were also robust and time-dependent (Addy, 2012).

Overall, the above controlled studies in humans confirm the basic pharmacology of salvinorin A in humans as a potent and fast-acting high efficacy KOPr agonist, as previously determined in rodent and non-human primate models.

## Salvinorin A as a Novel Scaffold for KOPr Medicinal Chemistry and Pharmacotherapy of Neuropsychiatric Disorders

As mentioned above, salvinorin a is a non-nitrogenous diterpene, and provides a new scaffold for development of new KOPr ligands, by semi-synthetic methods (Beguin et al., 2012; Prisinzano, 2013; White et al., 2015). Salvinorin A *per se* has several effects that complicate its use as a pharmacotherapeutic tool in humans, including dissociative and hallucinogenic effects, thought to be due to high efficacy KOPr agonism (Johnson et al., 2011; Ranganathan et al., 2012; MacLean et al., 2013). Salvinorin A also has extremely rapid onset and relatively short duration of action in humans and non-human primates, possibly mediated in part by the p-glycoprotein blood-brain barrier efflux transporter (Hooker et al., 2008; Butelman et al., 2009, 2012a; Johnson et al., 2011), and such rapid on/off effects further complicate its study in humans.

Medicinal chemistry efforts focusing on semi-synthetic salvinorin A analogs have resulted in some compounds with a differential *in vivo* profile compared to the parent neoclerodane (salvinorin A). These differential profiles may be due to a more gradual onset/offset (Wang et al., 2008), or to pharmacodynamic factors, including KOPr partial agonism, or differential signaling through downstream pathways (“biased agonism”; Zhou et al., 2013a). For example, some analogs, including 16-Br-salvinorin A and Mesyl-salvinorin B were able to decrease reinstatement of cocaine self-administration in rats, with a decreased burden of sedative-like effects, as caused by salvinorin A or by reference KOPr agonists (Riley et al., 2014; Simonson et al., 2014).

Salvinorin A and some common reference KOPr agonists (such as U69,593 and (-)-U50,488) have been classified as “unbiased” KOPr ligands, that is, they activate the adenylyl cyclase and β-arrestin pathways with approximately equal potency and efficacy (White et al., 2014). Of interest, 22-thiocyanatosalvinorin A (RB 64), a “biased” ligand (with greater potency at the adenylyl cyclase pathway compared to the β-arrestin signaling pathway), was able to produce antinociception in mice, with a reduced tendency to cause some undesirable effects such as locomotor incoordination in the rotorod assay (White et al., 2014, 2015). These efforts point the possibility of novel salvinorin-based neoclerodanes with therapeutic-like effects in preclinical models of neuropsychiatric states (including addictions and pain), with a reduced burden of “on-target” undesirable effects known to be caused by salvinorin A, or by classic KOPr agonists.

## Summary and Main Points of Consideration for Future Research

(a) Salvinorin A is a unique and potent plant-derived hallucinogen, and is a short-acting high efficacy KOPr agonist.
(b) KOPr (encoded by gene *OPRK1*) are present in several areas of the CNS and periphery, and are activated by their endogenous agonist neuropeptide ligands, the dynorphins (encoded by gene *PDYN*). KOPr/dynorphin systems are one of the major counter-modulatory systems to dopaminergic activation, in striatal pathways known to mediate motor activity, arousal and responses to natural and drug reinforcers. KOPr/dynorphin systems are also present in areas involved in mood, anxiety and stress processing (including amygdala and hypothalamus).
(c) Exposure to stress or to drugs of abuse (e.g., cocaine and other psychostimulants) results in an upregulation in KOPr/dynorphin activity, and this may underlie, in part, neurobiological and behavioral effects observed in these conditions.
(d) KOPr agonists including salvinorin A have characteristic effects in preclinical models, and cause anhedonia, depressant-like and sedative-like behaviors. These behaviors may in part be due to the KOPr-agonist induced counter-modulation of dopaminergic brain systems (mentioned above).
(e) The prominent salvinorin A-induced hallucinogenic effects implicate the KOPr/dynorphin system in higher cognitive and perceptual functions, in health and disease.
(f) Salvinorin A is a novel scaffold for medicinal chemistry approaches, with the goal of generating novel compounds with pharmacotherapeutic potential, in neuropsychiatric diseases, including specific addictions.

### Conflict of Interest Statement

The authors declare that the research was conducted in the absence of any commercial or financial relationships that could be construed as a potential conflict of interest.
