# Corrigendum: Salvinorin A, a kappa-opioid receptor agonist hallucinogen: pharmacology and potential template for novel pharmacotherapeutic agents in neuropsychiatric disorders

**DOI:** 10.3389/fphar.2015.00285

**Published:** 2015-11-30

**Authors:** Eduardo R. Butelman, Mary Jeanne Kreek

**Affiliations:** Laboratory on the Biology of Addictive Diseases, The Rockefeller UniversityNew York, NY, USA

**Keywords:** kappa-opioid receptor, dynorphins, salvinorin A, *Salvia divinorum*, depression, addiction

Reason for Corrigendum:

In the original review, we placed an incorrect citation in the following sentence:

“Also, salvinorin A can act as a “punisher” to self-administration of cocaine or a short-acting MOPr agonist in primates (when given contingently; Whitfield et al., 2015).”

The correct sentence and citation are as follows:

“Also, salvinorin A can act as a “punisher” to self-administration of cocaine or a short-acting MOPr agonist in primates (when given contingently; Freeman et al., 2014).”

The authors apologize for the mistake. This error does not change the scientific conclusions of the review.

## Conflict of interest statement

The authors declare that the research was conducted in the absence of any commercial or financial relationships that could be construed as a potential conflict of interest.
